# LARS: A Light-Augmented Reality System for Collective Robotic Interaction †

**DOI:** 10.3390/s25175412

**Published:** 2025-09-02

**Authors:** Mohsen Raoufi, Pawel Romanczuk, Heiko Hamann

**Affiliations:** 1 Science of Intelligence, Research Cluster of Excellence, 10587 Berlin, Germany; pawel.romanczuk@hu-berlin.de (P.R.); heiko.hamann@uni-konstanz.de (H.H.); 2 Department of Electrical Engineering and Computer Science, Technical University of Berlin, 10587 Berlin, Germany; 3 Department of Biology, Humboldt University of Berlin, 10115 Berlin, Germany; 4 Department of Computer and Information Science, University of Konstanz, 78457 Konstanz, Germany

**Keywords:** collective robotics, swarm robotics, multi-robot systems, Extended Reality (XR), Augmented Reality (AR), Mixed Reality (MR), open-source robotics, human–robot interaction, educational robotics

## Abstract

Collective robotics systems hold great potential for future education and public engagement; however, only a few are utilized in these contexts. One reason is the lack of accessible tools to convey their complex, embodied interactions. In this work, we introduce the Light-Augmented Reality System (LARS), an open-source, marker-free, cross-platform tool designed to support experimentation, education, and outreach in collective robotics. LARS employs Extended Reality (XR) to project dynamic visual objects into the physical environment. This enables indirect robot–robot communication through stigmergy while preserving the physical and sensing constraints of the real robots, and enhances robot–human interaction by making otherwise hidden information visible. The system is low-cost, easy to deploy, and platform-independent without requiring hardware modifications. By projecting visible information in real time, LARS facilitates reproducible experiments and bridges the gap between abstract collective dynamics and observable behavior. We demonstrate that LARS can serve both as a research tool and as a means to motivate students and the broader public to engage with collective robotics. Its accessibility and flexibility make it an effective platform for illustrating complex multi-robot interactions, promoting hands-on learning, and expanding public understanding of collective, embodied intelligence.

## 1. Introduction

Since the first appearance of Extended Reality (XR) technologies, they have been adopted across a wide range of applications [1,2,3,4]. Among these, Augmented Reality (AR) and Mixed Reality (MR), in particular, are increasingly used in educational technology and robotics [5,6]. These tools enrich learning experiences and broaden research possibilities. In education, XR facilitates understanding of abstract or complex topics through immersive and interactive experiences [2]. In robotics, and especially in multi-robot systems, XR offers significant potential for education, providing opportunities to showcase collective behaviors and engage a general audience in an instructive and interactive manner. In these systems, XR tools create dynamic virtual environments that are observable to humans and serve as a medium for interaction with multi-robot systems. By making intangible and complex concepts in collective systems more accessible to human users, XR technologies support better understanding, analysis, and communication of how these complex systems function.

The application of XR technology in robotics research goes further than simple visualization; it becomes a basis for developing and testing advanced multi-robot scenarios [5,7]. They open up new possibilities for researchers to study their system in augmented environments that go beyond the real-world limitations of lab settings. At the core, these systems are equipped with detection and tracking components which are necessary tools to study physical collective systems, whether artificial or natural [8,9]. As an intermediate step between simulation and real-world deployment, XR platforms can split the sim-to-real gap into a sim-to-lab and a lab-to-real step, making bridging the gap toward realization more manageable.

We present the Light-Augmented Reality System (LARS) as an open-source, and cost-effective AR tool for collective robotics. LARS uses projection-based augmentation to embed virtual elements directly into the robots’ shared physical environment, enabling indirect robot–robot and human–robot interaction without modifying the physical robots. It is compatible with a range of mobile robots, from miniature platforms (e.g., Kilobot [10]) to mid-sized robots (e.g., Thymio [11]).

LARS has been developed and refined through its use in multiple collective robotics projects [12,13], evolving to meet the needs of research in this field. Beyond research, LARS has also been used in public demonstrations to make complex concepts interpretable to human observers through visual augmentation. These experiences shaped a set of design requirements for creating a robust, versatile tool for collective robotics research, education, and outreach.

**Figure 1:**
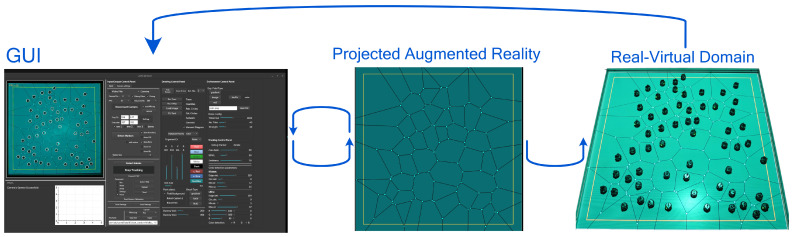
The view layer presents two main interfaces: the GUI (the left window) which contains the bird’s-eye view of the camera, with overlaid rendered information (such as FPS and robot IDs), and the virtual scene (the middle picture), which is projected as the second display on the environment. The right image shows the real–virtual domain, containing both the robots and the virtual visual objects such as the Voronoi tessellations of the space.

Based on these experiences, we listed a set of design requirements that guided the development of LARS. The key requirements are as follows:

- R1: Closed-loop interaction. The system must support experiments where the virtual environment, and hence visual feedback, is continuously updated based on the tracked state of robots, running as a standalone setup without requiring external infrastructure (See Figure 1).
- R2: Platform adaptability. The system must support marker-free tracking across diverse robotic platforms, enabling integration with different robot types and simplifying experimental setup.
- R3: Low-latency response. The system should maintain low latency and perceptually smooth visual updates to ensure accurate timing in interactive tasks.
- R4: Flexible environment augmentation. Users should be able to project dynamic, versatile elements, such as gradients, or visual noise, that both robots and human observers can interpret.
- R5: Runtime configurability. Parameters (e.g., detection thresholds) must be adjustable at runtime via the GUI, without restarting the software or modifying source code.
- R6: Integrated data logging. The system should provide built-in data logging and video recording for post-experiment analysis.
- R7: Scalability and efficiency. The architecture should remain computationally efficient while supporting large numbers of agents.
- R8: Open accessibility. LARS is released as a free and open-source tool, explicitly building on prior open-source software and extending this collaborative practice to support transparency, reproducibility, and community-driven development [14].

## 2. Related Work

### 2.1. Augmented and Extended Reality Systems

Extending reality by augmenting virtual objects into the physical world has been widely studied both as an industrial technology and as a research tool [2,3,15]. Extended Reality refers broadly to technologies that blend real and virtual content. This includes Augmented Reality, where virtual elements are overlaid onto the real world; Mixed Reality, where real and virtual elements coexist and interact; and Virtual Reality, which immerses the user entirely in a synthetic environment [16,17,18]. While these terms are sometimes used inconsistently [2], they span a continuum of immersion, ranging from minimal augmentation of the real world to fully virtual experiences. Most XR systems are developed for human users, accessed through head-mounted displays, tablets, or smartphones. However, robots have also been considered as target users in several studies [7,19]. The applications of XR systems in robotics cover a wide range of topics, mainly with the aim of enhancing HRI with the focus on the projected information observable only for the human user [7,20,21]. Unlike humans, robots do not rely on visual interfaces but instead perceive their environment through sensors. Consequently, XR systems designed for interaction with humans *and robots* require a spatial, environment-level approach to augmentation, rather than device-centric rendering.

### 2.2. Spatial Augmented Reality for Multi-Robot Systems

Spatial Augmented Reality (SAR) shifts the augmentation into the environment, typically via projectors or shared screens [22,23,24]. Instead of delivering content through wearable devices, SAR embeds virtual elements into the physical workspace, making them visible to both robots and humans without individual displays. In industrial robotics, SAR has been used to project instructions, task boundaries, or robot trajectories onto workspaces, enhancing human understanding and streamlining collaborative workflows without the need for screens or head-mounted displays [5,25].

However, the use of SAR in multi-robot systems, particularly in collective robotics, remains limited. One of the earliest examples [26] visualized pheromone trails projected into the environment to help interpret foraging behaviors. While effective for observation, this system was tailored to a specific experiment and lacked general-purpose capabilities or support for robot–environment interaction. In contrast to the SAR approach, a few systems have adopted a Virtual Reality approach for multi-robot systems, in which the physical limitations of the robots are modified or extended virtually. For example, in Kilogrid [27] and ARK [28,29], robots operate in an environment that is digitally controlled, with virtual cues such as gradients or pheromone fields projected into the arena. These systems enable precise control of swarm behavior through environmental modulation, but they are often designed for a single platform and are not easily transferable to other robots or experimental designs. In essence, they shift the system toward the VR end of the XR spectrum, focusing more on digitally defining the robot’s world than on making physical environments more interpretable or interactive.

Compared to prior XR-based systems for multi-robot platforms, LARS aims to generalize beyond single-purpose tools by integrating multiple functions into a unified, flexible architecture. Previous systems have often focused on one or two specific objectives: some provided real-time logging or experiment replay capabilities [30,31]; others visualized robot states or internal variables on a GUI to support analysis [5,32,33] or through an AR headset for human users [34]; some enabled limited virtual environmental features to test behaviors under specific conditions [26,35,36,37]; and a few extended robot capabilities using platform-specific augmentation [27,28,38].

Unlike these, LARS is designed as a general-purpose SAR system that combines these objectives in a closed-loop setup by projecting virtual elements directly into the physical arena, creating a shared real–virtual environment visible to both robots and human observers. It supports marker-free tracking across various robot types, enables spatial interaction through dynamic visual cues, allows control over virtual environmental properties, and embeds visualization directly into the shared physical space. These features make it suitable for scalable, interactive, and observational experiments in collective robotics. A detailed comparison with prior systems is provided in Table 1.

### 2.3. Tracking and Localization Systems

Many tracking systems have been developed to study collective behavior in biological and artificial systems. In biology, tracking enables quantitative analysis of group-level patterns, while in robotics, it supports experiment documentation and performance evaluation. Most existing systems rely on physical markers or tags to identify individuals [39,40,41,42], but marker-free approaches offer greater flexibility [43], especially for offline post-processing of recorded videos.

For online tracking, marker-free systems face added challenges, as they require prior knowledge of object properties (e.g., shape or appearance). In tracking multiple objects, they must also maintain accuracy and identity consistency across scales. Choosing the appropriate tracking approach involves navigating trade-offs, such as generalization versus specialization. In the design of our tracking system, we take advantage of the geometric regularity of the robots: from a downward-facing camera, their bodies appear approximately circular. This allows us to adopt an algorithm originally developed specifically for Kilobots, ARK [28], which detects robots using simple circle detection. When combined with more sophisticated filtering and tracking algorithms, this minimal detection approach proves robust in maintaining individual identities, even during close interactions and collisions. For non-circular robots, approximate roundness is sufficient; alternatively, a simple printed ring can also be added to improve detection accuracy. This design enables us to robustly track a variety of mobile robots, as we demonstrate later in the paper.

## 3. Materials and Methods

The core material of this work is the LARS system itself, which consists of a software–hardware pipeline for tracking, visualization, and interaction in collective robotics experiments. In this section, we first describe the software architecture that coordinates these functions, followed by details of its implementation.

### 3.1. Software Architecture

The goal of the software is to provide an all-in-one application, running on a local computer connected to the camera and projector, that orchestrates the coordination between these components along with user inputs received either through a GUI or directly from a controller device. The application uses a predetermined physical model to generate virtual, visual scenes that interact with robots. A projector displays the scene directly onto the surface where the robots are placed. This interface serves as the primary means to merge virtual and real-world elements into a Mixed Reality world. LARS also includes interfaces that use central information to transmits messages directly to the robots. Although the use of a central transmitter, such as the overhead controller for Kilobots, departs from the decentralized assumptions and bypasses the physical limitation of robots, we keep this feature because it allows users to program the robots and send control commands, such as starting or stopping experiments.

**Figure 2:**
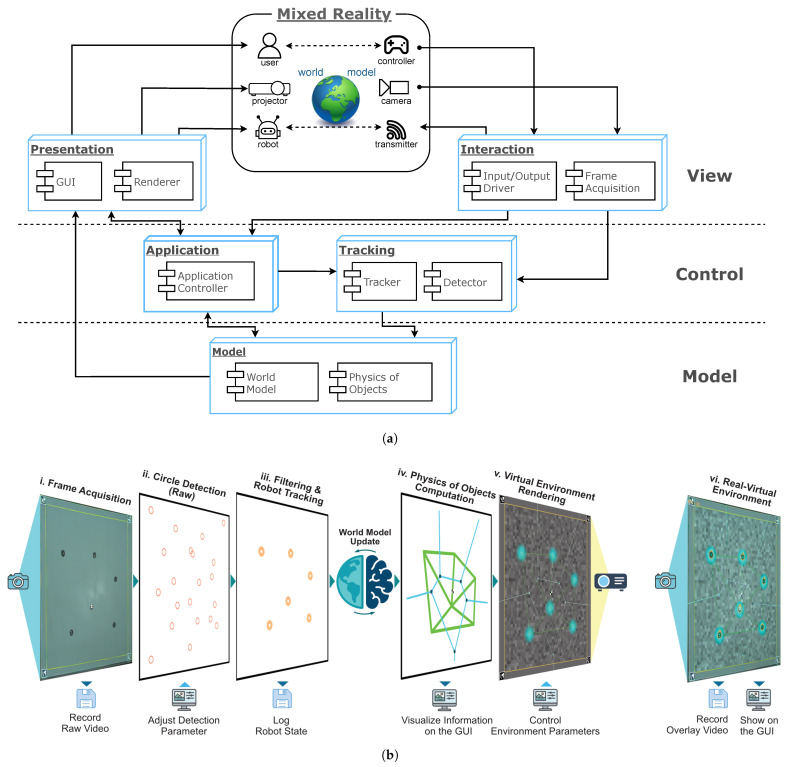
(**a**) LARS software and hardware architecture structured into model, control, and view layers. The pipeline forms a closed loop from sensing (camera) to computation (detection, tracking, world model, physics) to actuation (projection), with user interaction via the GUI and optional robot controller, enabling interaction between users, robots, and the augmented environment. (**b**) Schematic runtime workflow for one update cycle: (i) frame acquisition; (ii) circle detection (raw detections, including noise and false positives); (iii) tracking with filtering and data association to maintain consistent robot IDs; (iv) world model update and environment dynamics, including interaction networks, communication fields, and other virtual cues; (v) rendering via Qt Painter and projection mapping; (vi) updated real–virtual environment as presented in the next camera frame to robots and users. Logging and video recording run in parallel, while the GUI allows users to monitor system state and adjust parameters at runtime.

By incorporating insights from established software design paradigms [30], LARS adopts and extends the Model–View–Controller (MVC) design pattern [44,45] to address the design requirements outlined in Section 1 (R1–R7). This separation between the world model, control logic, and visualization allows each layer to be modified or replaced independently, simplifying adaptation to new platforms (R2) and experimental needs (R3–R7). The MVC pattern is thus well-suited to facilitating immersive interaction between users (human and robot) and the virtual and physical elements in the arena (see Figure 2).

#### 3.1.1. View Layer

The view layer manages the system’s visual representation and user interaction. It consists of two main components:

- *Presentation Block* is responsible for displaying the XR environment and presenting information to the user. It includes the GUI, which provides an enhanced view of the environment from the bird’s-eye camera with augmented overlays tailored to the experiment’s objectives (R4).
- *Interaction Block* manages both human and robot inputs. Users interact with the system either indirectly via GUI functions or directly by sending messages to the robots (R5). This block also acquires visual data from the camera and forwards it to the tracking module in the control layer.

#### 3.1.2. Control Layer

The control layer handles the operational logic of the application. It consists of the following:

- *Application Controller Module* governs execution flow, coordinates between modules, processes user and system events, and updates the world model based on scene changes (R1).
- *Tracking Block* detects and tracks objects in the environment to ensure consistent identification across frames (R2).

#### 3.1.3. Model Layer

The model layer contains the internal representation of the XR environment. It includes the world model, which stores the current state of the environment, and the Physics of Objects module, which defines the physical rules for virtual entities (R1, R4).

### 3.2. Implementation Details

We implemented the software in C++ language and based the application in the Qt framework [46], using the OpenCV library [47] for image processing and real-time video handling. These choices are motivated by their open-source nature, strong community support, and suitability for real-time performance. The current version of LARS is tested on Ubuntu 20.04 and 22.04 (R8).

The view layer’s Presentation Block is implemented using Qt Painter for rendering virtual elements such as shapes, gradients, noise textures, and overlays (e.g., Voronoi diagrams, interaction networks). Rendering is updated every 15 ms (60 FPS) via a timer-driven loop, with the output sent to the projector as a secondary display. Spatial alignment between the virtual scene and the physical arena is achieved through the virtual–real coordinate mapping described in Section 3.4.

In the control layer, synchronization between tracking, rendering, and projection is managed within Qt’s main event loop, ensuring that each projected frame corresponds to the most recent tracking data (R3). Because LARS is a standalone, closed-loop system that does not rely on external tracking data, or direct, real-time state exchange with robots, strict hardware-level synchronization is not strictly necessary. Frame-to-frame consistency is sufficient for both robot sensing and human observation, allowing the system to remain lightweight while maintaining responsive interaction.

### 3.3. Detection and Tracking Infrastructure

The detection and tracking module in LARS builds upon the tracker developed for ARK [28], which has been tested effectively for Kilobot experiments. Leveraging the generality of the detection based on the Hough circle method implemented by CUDA libraries [48,49,50], and harnessing the regularity of circular shape of swarm robotic platforms, LARS extends the application to any circular-shaped robot, beyond Kilobot (R2). While full circularity is not required, detection remains effective for platforms like Thymio II with semi-rounded shapes. For non-circular platforms, a simple printed ring can be added to improve detection robustness. By optimizing some parts of the original ARK code, we boosted the tracking performance from 9 to 38 FPS in the current version of LARS (R3, R7). For more technical details, please see the repository (original implementation: https://github.com/DiODeProject/KilobotArena, accessed on 22 August 2025 [28]; our modified version: https://github.com/mohsen-raoufi/LARS, accessed on 22 August 2025 [51]).

In addition to position, the system detects robot colors, which can represent internal states (e.g., LED indicators on Kilobots). To improve reliability, color detection is currently limited to red and blue, as demonstrated in synchronization [13] and heterogeneous phototaxis scenarios [12].

### 3.4. Mapping Virtual to Real Coordinates

**Figure 3:**
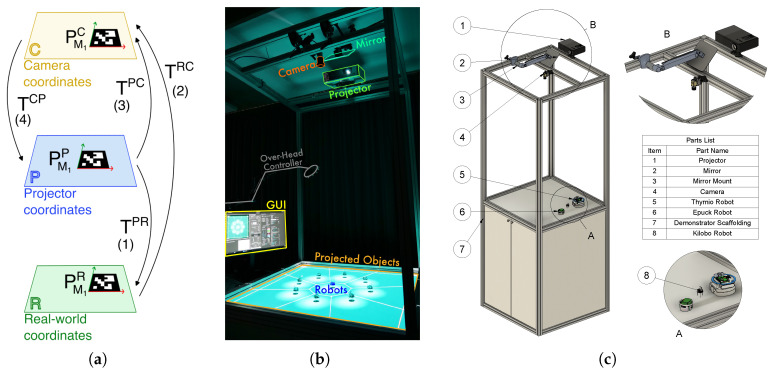
(**a**) Mapping and translation between virtual and real coordinates by using ArUco markers. (**b**) Real Kilobot demonstrator using LARS showing nine Kilobots detected in an environment, with light projection at their detected locations, and the visualization of Voronoi tessellation. (**c**) The 3D model of the full demonstrator including the hardware structure with scaffolding, camera, projector, and mirror. The mirror increases the effective projection distance, compensating for the projector’s limited projection angle and enabling full coverage of the robot arena.

To connect the virtual world to the real environment, we need to find the relation between three coordinates: projector (P), real-world (R), and camera (C) (as shown in Figure 3a). This process also makes self-calibration possible and ensures precise projection of virtual objects into the real world with every execution of the application. We use four Fiducial (ArUco [52]) markers, with predetermined positions in the projector coordinates (e.g., $P_{M_{1}}^{P}$). The user determines the locations based on the desired location of the arena. The arena specifies the boundary within which the experiment is conducted (see Figure 1). Once all four markers are detected on the camera frame ($P_{M_{1}}^{C}$), we apply a getPerspectiveTransform function [47] to calculate the transformation matrix from the P to C coordinates ($T^{\mathrm{PC}}$). The inverse of this matrix provides the transformation in the opposite direction ($T^{\mathrm{CP}}={\left(T^{\mathrm{PC}}\right)}^{-1}$). This method allows us to project virtual objects into the physical world without needing their explicit position in the real-world coordinates. The transformation matrix $T^{\mathrm{CP}}$ is the key to mapping virtual objects onto the real world. Given the position of objects (e.g., robots) in the camera frame and the transformation matrix, the program ensures seamless interaction between the entities of the two worlds.

### 3.5. Augmenting Reality with Projected Visual Objects

Modifying the environment based on the state of robots enables the possibility of implementing indirect communication among robots, between robots and the environment, and between robots and human users. To achieve this, we augment the environment by creating virtual scenes. Creating virtual scenes for XR environments for human users typically involves very intricate processes to make the integration of the virtual entities into the real world seamless, and enhance the user’s interactive experience. Our objective in determining the appropriate level of complexity for virtual object design was to eliminate excessive nuances that do not enhance the quality of experience for either human or robot users. This simplification reduces computational load and enhances overall system performance.

At the base of creating projected visual objects, we rely on the WM which encompasses the fundamental principles of physics governing the interaction between objects in the XR environment, as well as the parameters and states of the world. The user defines the rules according to the requirements of the experiments in the WM. Consider a setting in which the trace of robots’ movement resembles pheromone trails. The aggregation of pheromones is modeled via the principle physical rules of the WM. Additionally, environmental parameters including the evaporation rate and the width or intensity of the pheromone are described in the WM.

### 3.6. Direct Communication to Robots

Compared to indirect augmented communication, which presumes the realistic limitation of robotic platforms, a direct and centralized communication approach allows researchers to explore system behavior in configurations that exceed its physical restrictions. This freedom in experimentation has contributed to the adoption of this approach in various previous studies. To preserve this direct interaction capacity, we implemented a channel for sending commands to the robots via output devices. However, it is important to note that this aspect is not the primary focus of this paper. In our experiments with two platforms, we utilized the infrared (IR) messaging protocol via the overhead controller (OHC) for Kilobots, and WiFi communication for other more advanced robots (e.g., Thymios with Raspberry Pi board, Raspberry Pi Foundation, Cambridge, UK). Combining this centralized communication with the projection of the network (e.g., a Voronoi-based network) the user can effectively demonstrate an abstract notion in a tangible way. The benefit of integrating an output device controller specifically for Kilobots is to program the robots via the OHC. In addition, it enables immersing the reality with more virtual information, as demonstrated in [28]. This enables robots to interact with a virtual environment, where messages are sent to the robots directly.

## 4. Results

To assess the capabilities of LARS, we conducted a series of benchmarking experiments that measure the system’s performance under different conditions. These include tests of scalability, tracking precision, responsiveness under processing load, and robustness to environmental noise and lighting variations. With these benchmarks, we establish that LARS is both reliable and efficient, even at collective scales and under various environmental conditions, such as brightness or visual noise.

### 4.1. Tracking Precision with 100 Stationary Real Robots

To assess our system’s real-world tracking performance, we placed 100 real Kilobots in a known grid pattern. Then we tracked and logged their positions for two minutes. In this experiment, we measured tracking precision and frame rate performance of the system under two processing conditions: (1) logging only, where positions were recorded to a text file without saving any video; and (2) logging plus video recording, where both the raw camera input and the processed GUI frames were saved as video files in real time (R6).

**Figure 4:**
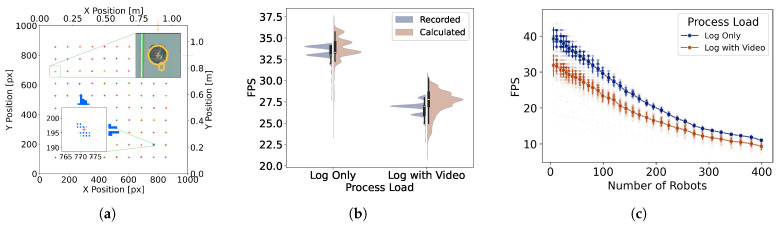
Evaluation of tracking precision, system performance, and scalability. (**a**) Tracked position of 100 real Kilobots, with different color for each robot. The lower inset shows the distribution of the tracked position for the robot with highest tracking error (i.e., highest variance). The red plus sign marks the average tracked position (gray dots). The upper inset shows the detected points overlaid on an image of the actual Kilobot. (**b**) Comparison of recorded and calculated FPS under two processing conditions. Video recording reduces performance, while both FPS measures remain consistent across loads. (**c**) Scalability test showing FPS as a function of the number of robots. FPS decreases with increasing swarm size, but the system remains responsive even with 399 robots, maintaining approximately 10 FPS under the heavy processing load. Each data point is averaged over multiple trials, with error bars indicating standard deviation.

To evaluate tracking precision, we measured the standard deviation of each robot’s detected position over time while the robots remained stationary. The resulting distribution of position noise was low across all robots, confirming that LARS maintains accurate tracking even under moderate processing loads (see Figure 4a). We also did not observe any spatial correlation for the precision error. Frame rate (captured by FPS) was computed both from software-reported values and from timestamp differences in the log files, with negligible discrepancy between the two, indicating consistent and reliable timing (see Figure 4b). Considering 30 FPS as a baseline for smooth tracking and visualization performance, the performance is higher than this threshold for the log-only case but falls slightly below it under higher processing loads. Next, we characterize performance scalability with respect to the number of tracked objects.

### 4.2. System Scalability

In this experiment, we evaluated how tracking scales with an increasing number of robots (R7). To be able to go beyond the limited number of physical robots in our lab, we rendered projections of up to 399 virtual robots. Each robot was rendered in the scene and tracked using the standard pipeline. The robots were arranged in grid formations of varying dimensions, and we measured the system’s performance under two processing conditions as before.

The system maintained stable performance with up to 399 robots, with FPS decreasing gradually as the number of tracked objects increased. Even under heavier processing load (videos recording plus logging), it remained usable in most scenarios, confirming scalability beyond typical lab-scale experiments. It is notable, in the log-only condition, that the system could track 200 robots with a rate of 20 FPS which, to the best of our knowledge, exceeds the scale of current lab experiments with real robots. In the extreme case of 399 virtual robots, the system performed at around 10 FPS representing an improvement compared to the closest related work [28].

### 4.3. Robustness to Environmental Conditions

To evaluate the robustness of LARS under varying environmental conditions, we conducted two systematic experiments using 100 real Kilobots arranged on a stationary grid. The goal was to quantify how detection and tracking performance respond to changes in (i) lighting and (ii) spatial visual noise.

#### 4.3.1. Lighting Conditions

**Figure 5:**
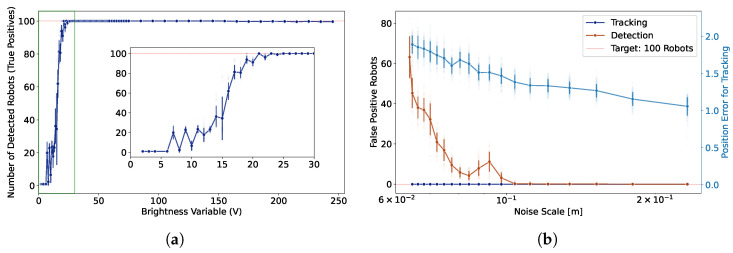
Evaluation of detection and tracking robustness under brightness and visual noise conditions. (**a**) Number of correctly detected robots (true positives) as a function of the brightness variable (V) of the environment in HSV space. The actual number of robots in the environment is 100 (red dotted line); values below this indicate missed detections. Detection performance declines sharply below a brightness threshold (V≈30). The inset shows a zoomed view of the low-brightness regime (green rectangle). (**b**) Robustness to visual noise projected into the environment. Left axis: number of false positives in detection and tracking across varying spatial scales of Gaussian noise. Right axis: mean positional error in tracking. While detection accuracy decreases at finer noise scales, tracking maintains correct count with only a moderate increase in spatial error. For both plots, each data point is averaged over multiple trials, with error bars indicating standard deviation.

In the first experiment, we varied the brightness of the projected white background and observed its effect on the detection module. Since precise light measurements (e.g., in lux) were unavailable, we used the value (V) component in the HSV color space as a proxy for brightness. Starting from maximum brightness (V = 255), we gradually reduced the value to zero. At each level, the detection procedure was repeated 50 times using an automated routine implemented in LARS, without involving the tracking component. As shown in Figure 5, LARS maintained perfect detection (100 true positives) above a brightness threshold of approximately 30, which is a dark setting (see the Supplementary Video S1 [53]). Below this range, performance degraded sharply, with increased variance across repetitions. This drop-off highlights the importance of adequate contrast between the robots and the background in enabling reliable marker-free detection.

#### 4.3.2. Environmental Visual Noise

In the second experiment, we tested the effect of structured visual noise on detection and tracking performance. We generated a dynamic Gaussian noise field and projected on top of the robot arena, and its spatial scale was systematically adjusted by varying the number of tiles used to discretize the noise field. We started from large tiles (2 × 2) with high spatial correlation to finer resolutions. Here, we evaluated both detection-only and tracking modes.

As shown in Figure 5, the number of false positives in detection increases significantly at fine noise scales, particularly when the spatial correlation of the background decreases. In contrast, the tracking module maintains a fixed count of 100 robots by construction and shows no false positives, but the positional accuracy degrades under higher visual noise. We quantified this degradation by computing the mean positional error of the tracked robots compared to their positions in noiseless condition. While the error increases at smaller noise scales, the absolute values remain low, confirming that tracking remains stable even under degraded visual conditions. The Supplementary Video S1 demonstrates this robustness qualitatively [53].

Together, these experiments demonstrate that while the detection pipeline is sensitive to lighting and visual noise, the tracking module is comparatively more robust in both count and spatial accuracy. These findings underscore the value of initializing tracking under clean conditions.

### 4.4. Closed-Loop Latency

In systems like LARS, where visual tracking is tightly integrated with real-time projection, evaluating closed-loop latency is essential. Unlike conventional tracking-only benchmarks, the end-to-end latency includes delays introduced by image acquisition, processing, decision-making, and rendering to the physical environment. Therefore, we conducted an experiment to characterize the full latency of the system from image acquisition to detection and tracking to projection, as experienced through the projected visual feedback (R3).

**Figure 6:**
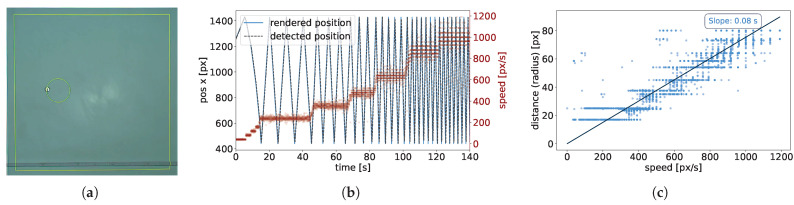
Evaluation of closed-loop latency in the perception-to-rendering pipeline. (**a**) Experimental setup showing a virtual robot (with an image of a Kilobot) moving leftward while a projected ring (yellow) lags behind due to system latency. The ring’s radius was manually adjusted so that the robot’s center aligned with the edge of the ring. (**b**) Position and speed of the virtual robot over time during the latency evaluation experiment. The blue curve shows the oscillatory x-position of the robot as it moves back and forth across the arena, while the red steps indicate the corresponding speed values manually configured during the experiment. (**c**) Measured radius of the ring (i.e., spatial lag) as a function of the robot’s speed. The slope of the linear regression indicates an approximate latency of 0.08 s. Each point corresponds to a manual measurement at a given speed.

We designed an experiment using a virtual robot moving in a straight-line trajectory. The virtual robot was rendered and updated continuously within the system, moving back and forth across the arena between its boundaries. Its speed was varied systematically across trials. Simultaneously, a yellow ring was projected at the robot’s tracked position to visualize the delay between the computed location and the projected output. Due to processing and rendering latency, a spatial offset emerged between the robot’s actual position and the center of the projected ring (see Figure 6a). This offset represented the delay in the closed-loop pipeline and increased with robot speed. To quantify this delay, we manually adjusted the radius of the ring throughout the experiment such that the virtual robot’s center approximately coincided with the edge of the ring. Although this process introduced some measurement uncertainty, it provided a practical way to estimate the spatial gap between robot and ring under different motion speeds. A plot of the recorded ring radius versus the robot’s speed shows a clear linear relationship (Figure 6c), with a regression slope of 0.08 s, which we interpret as the effective end-to-end latency of the system.

As an additional check, we analyzed a video recording of the experiment (see the Supplementary Video S1 [53]) and compared the temporal period of the robot’s oscillatory motion with that of the projected ring. The time difference between these two signals was consistent with the spatial measurement and yielded a latency estimate in the range of 0.05–0.06 s.

**Figure 7:**
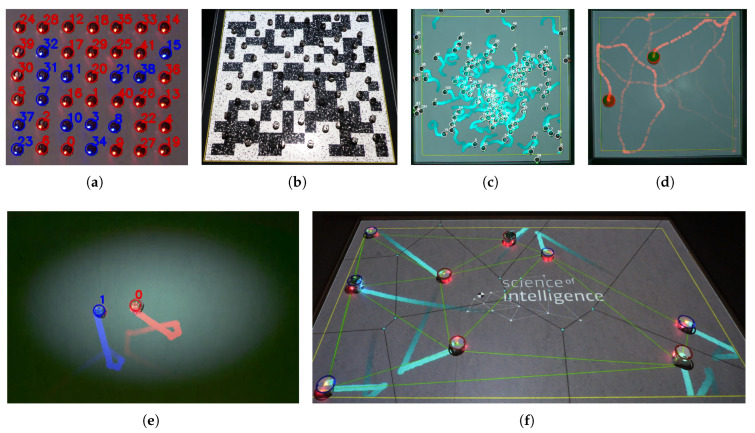
Example scenarios with different robots and environment settings. (**a**) GUI snapshot of 42 Kilobots synchronizing on a grid with their internal binary state being detected by the color of their LED in blue or red; (**b**) user view of 63 Kilobots making a collective decision on a tiled environment with projected dynamic salt-and-pepper noise [54]; (**c**) GUI snapshot of 109 Kilobots with the trace of their random movement decaying over time; (**d**) GUI snapshot of two active balls randomly moving in the bounded arena, being tracked by LARS without the need for any markers; (**e**) GUI snapshot of two Thymios with different colors locating the center of the light distribution (projected by LARS). The trace of each robot shows the consistency of the color detection of each robot over time, even after a collision [54]. (**f**) User view of Thymios moving randomly, with their centroid, the projection of their trajectory (light blue trails), their Voronoi tessellation (black lines) and the corresponding network (green lines).

It is important to interpret latency in the context of the experiment being performed. For instance, in slow-moving systems such as Kilobots, even relatively high latencies may still be acceptable, as the robots’ low speeds make them more tolerant to visual feedback delays. Furthermore, in scenarios with higher processing demands, such as complex visual overlays, real-time logging, or larger collectives, we expect an increase in latency, similar to what we observed for the reduced FPS. Nonetheless, the latency observed in our tests remains within a practical range for most collective robotics experiments. For example, in a demonstration with nine Thymio robots (Figure 7f), the projected traces remain closely aligned with the robots’ positions, with no visible lag between the robot bodies and the endpoint of their motion paths. This visual coherence provides a practical indication of the system’s responsiveness and confirms that the latency remains negligible relative to the temporal and spatial resolution required by the experiment.

## 5. Discussion

The effectiveness of an interactive system such as LARS can not be fully evaluated by benchmarks like tracking accuracy, latency, or calibration precision alone. While these metrics are essential for baseline validation, the broader utility of LARS lies in its ability to enable and support a broader range of experiments in collective robotics. By combining tracking, interaction, and visualization into a closed-loop single spatial Augmented Reality system, LARS facilitates experimental setups that would otherwise be difficult to implement in a typical laboratory setting. To show how these capabilities manifest in practice, we tested a set of use-case scenarios where LARS was deployed in varied experimental contexts. These scenarios demonstrate how the system meets the design goals of enabling light-based interaction, supporting marker-free tracking across robot platforms, and improving observability of complex collective behaviors. The main features enabling these capabilities are summarized below.

- **Light-Based Visualization and Interaction: **LARS uses projected light as a medium for indirect communication, enabling stigmergic coordination among robots and improving the observability of the system for human users (R1). It supports a range of static and dynamic visual elements, including programmable fields and noise patterns (Figure 7b), and overlays representing abstract properties such as interaction networks and centroids (Figure 7f).
- **Marker-Free Cross-Platform Tracking: ** The system supports marker-free tracking for a variety of ground robot platforms without requiring hardware modifications (R2). We demonstrated this capability with Kilobots (small), and Thymios (medium), as well as passive moving objects such as colored balls (see Figure 7d and the Supplementary Video S2 [55]). To adapt the detection to a new platform, the user adjusts only a few detection parameters (four sliders) via the GUI, without restarting the software or altering the code. This flexibility simplifies integration and reduces setup time across different experimental platforms.
- **Responsive Performance: **LARS maintains high frame rates and low latency, crucial for perceptually smooth performance for dynamic and responsive interactions in AR applications (R3). Our system on a desktop PC (housing a 16-core CPU, and 32 GB RAM) achieves a frame rate of around 38 frames per second (see Figure 4c for more details). This number exceeds the requirement for most laboratory scenarios involving ground mobile robots. The low-latency performance is evident from the projected traces closely following the robots’ movements without visible lag or mismatch (see Figure 7f, and the Supplementary Video S3 [56]).
- **Scalability to Robot Numbers: **LARS supports experiments at scale, with tested capacity reaching up to 109 real Kilobots (Figure 7c), and 399 (Figure 4c) simultaneously tracked and rendered virtual robots (R7). Additionally, it recorded a video stream on local storage and logged the position of each robot in a text file without a significant drop in the performance. The actual frame rate varied depending on concurrent processes, particularly file-writing operations, as explained in the previous section (see Figure 4b). This demonstrates the system’s ability to satisfy the requirement for lab experiment hardware with various collective sizes.
- **Integrated Logging and Capturing System: ** Besides all the online processes during an experiment, logging the data and recording the video frames are necessary features to enable post-processing and analysis of the experiment offline (R6). We implemented a logging system that records the state of robots, including their position, velocity, color, and IDs for every frame. In addition, two threads run to record the unprocessed frame data from the camera, and the processed frame data after tracking. The processed frame is similar to what is displayed to the user on the GUI.
- **Ease of Setup and Robustness: ** The self-calibration feature of the system enabled us to easily adjust the size of the arena from Thymios (3.7×2.3 m^2^, in Figure 7f) to Kilobots (1.0×1.0 m^2^, e.g., in Figure 7b) by simply modifying four inputs (R2). This user-friendly setup process eliminates complex configuration steps. The system’s ease of setup is combined with its robustness, which has been validated through continuous operation under demanding conditions during public demonstrations and various events, where LARS was used to showcase collective robotic scenarios.
- **Controllable Environment Parameters: ** Users have complete control over the XR environment, either prior to the execution, for example by modifying the physics of interaction codes, or during its runtime by modifying the properties of the environment via the GUI (R5).
- **Cost-Effectiveness: ** One of the main goals of releasing LARS is to provide accessibility to research tools by creating an affordable system that facilitates the use of XR technologies in developing laboratories, non-commercial users in academia, and educational institutions.

### Current Limitations and Future Directions

In the development of the system, our objective was to satisfy the requirements that apply to most general collective robotic experiments. While we have pushed the limits of what LARS can do, recognizing its constraints will clarify the necessary next steps for further development of the system. We list several LARS features that may require further development and customization to meet specific individual requirements of different users in the future.

- Explicit orientation detection: We use circle detection for the localization of robots in the environment. While an implicit orientation is calculated based on the direction of the velocity vector of robots, it is necessary to explicitly track the orientation of robots in some experiments.
- Reading tracking data from external trackers: Despite being designed for affordability, users might prefer to integrate marker-based tracker systems, like ViCon [57] or OptiTrack [58]. Such advanced tracking systems offer precise and high-frequency tracking data, including orientation and position in the three-dimensional space. Integrating this data into LARS will improve the performance of the tracking component.
- Interact with external programs for possible extensions: LARS is designed as a modular, standalone software that contains all the necessary functionalities. Nevertheless, incorporating the use of other programs will enhance its adaptability to meet more sophisticated requirements. In our evaluation experiments, we implemented an offline version of this approach by calling a Python (v3.9, 3.x series) function to create images or animations of environments containing robots with specific behaviors or parameters. An online approach, where the external program receives the state of the environment and generates the corresponding visual elements in real time for projection, remains a potential future extension.
- Offloading heavy environment generation to external programs: Stress testing revealed that extremely high-frequency, high-resolution environment generation (e.g., dense noise patterns) can slow down the system. In such special cases, offloading the rendering process to an external program and importing the resulting environment file into LARS could reduce computational load and maintain real-time performance.
- Flexibility to varying number of robots during an experiment: The current tracking algorithm assumes a fixed number of robots to track during the course of an experiment. However, some experiments may require adaptive tracking as the population size changes over time, although this introduces a trade-off in tracking accuracy. One potential solution is to define an upper limit on the number of robots and allow newly detected robots to be added dynamically if they pass the filtering process.
- More complex environments: Rendering more realistic virtual scenes is a primary goal in the development of AR for humans. Gamifying Mixed Reality with more complex and realistic objects will assist human user engagement, particularly for instructional applications.
- Extensive end-to-end benchmarking: While our evaluation includes scalability, latency, and robustness, a full benchmarking campaign would require assessing the system under a wider range of conditions, including moving real and virtual robots, occlusion scenarios, and long-term ID consistency. Compared to tracking-only evaluations, closed-loop SAR systems require well-defined end-to-end tests that are currently lacking in collective robotics research, and developing such benchmarks would advance both reproducibility and cross-platform comparability.

## 6. Conclusions

LARS is a testbed system designed to support multi-robot experimentation by combining tracking, logging, and visualization in a single, integrated platform. More importantly, it serves as a medium through which the environment is augmented with virtual objects, enabling new forms of interaction without altering the physical limitations of the robots. By projecting arbitrary visual elements into the arena, LARS enriches experimental conditions and expands the range of behaviors that can be studied. The augmented environment is a step to bridge the gap between simulation and reality.

Our benchmark evaluation demonstrates that LARS can scale to large numbers of robots, maintain tracking precision and responsiveness under different processing loads, and operate reliably under varying lighting and environmental conditions. Although not originally designed as a benchmarking tool, we showed that the system can be used to generate systematic performance data. We further illustrated the practical value of LARS through a range of example scenarios, involving both educational demonstrations and collective behavior experiments with Kilobots, Thymios, and passive objects. These examples highlight how LARS supports the design and communication of complex collective systems through light-based interaction and visual feedback. The videos of several demonstrations are available as Supplementary Material online (https://figshare.com/collections/LARS_videos/7995766/2, accessed on 22 August 2025).

The smooth projection of virtual observable elements alongside physical robots shows a powerful means to make collective robotics more accessible and interpretable. AR systems like LARS can be used to showcase complex collective behaviors in an intuitive way, making them particularly suitable for educational activities and public demonstrations. By expanding the range of possible experimental conditions without altering the physical limitations of robots, such tools provide a flexible interface for researchers to study these multi-robot system in controlled, repeatable, and visually enriched environments. As an open-source software (licensed under the GNU General Public License v3.0), LARS is available on this repository [51].

## Figures and Tables

**Table 1:** Related Extended Reality systems for multi-robots.

	FPS ^1^	Detection Method	Supported Robots [Tested on]	Immersion Level	Direct Commu- Nication	Max. Arena Size (m^2^)	Cost (K€)
**LARS**	38	Hough circle	Circular robots [Kilobot, Thymio, E-puck]	AR-MR with interactive visual objects	WiFi, IR for kilobot	2.3×3.7	1.5–4.0
ARK [28]	9	Hough circle	Circular robots ^2^ [Kilobots]	VR (only virtual)	IR for Kilobot	2.2×2.2	1.0–3.0
KiloGrid [27]	0.5 *	IR	Kilobots	VR (only virtual)	IR	1.0×2.0	≈15
ARDebug [30]	10 *	ArUco tags	Non-miniature robots	VR (only virtual)	WiFi, Bluetooth	2.5×2.5	1.0–3.0
[26]	5 *	Hough circle	Circular robots ^2^ [Alice]	AR with visual trails	N/A	1.4×1.05	1.5–4.0

^1^ (*) stands for the numbers reported by the reference. ^2^ The tracking algorithm can potentially be used on circular robots.

## Data Availability

The original data presented in the study are openly available in DepositOnce at https://depositonce.tu-berlin.de/handle/11303/25207, accessed on 22 August 2025. The updated version of the software can be accessed online via the GitHub repository at https://github.com/mohsen-raoufi/LARS, accessed on 22 August 2025.

## Supplementary Materials

The following supporting information can be downloaded at: https://www.mdpi.com/article/10.3390/s25175412/s1: Video S1: Benchmarking tests; Video S2: Platform Adaptability Test; Video S3: Introducing LARS with Example Behaviors.

## Abbreviations

The following abbreviations are used in this manuscript:

AR	Augmented Reality
ARK	Augmented Reality for Kilobots
AV	Augmented Virtuality
GPU	Graphics Processing Unit
GUI	Graphical User Interface
HRI	Human–Robot Interaction
HSI	Human–Swarm Interaction
IR	Infrared
LARS	Light-Augmented Reality System
MR	Mixed Reality
MVC	Model–View–Controller
OHC	Overhead Controller
WM	World Model
XR	Extended Reality
